# The complete mitochondrial genome of *Heniochus chrysostomus* (Perciformes, Chaetodontidae)

**DOI:** 10.1080/23802359.2021.1888335

**Published:** 2021-03-16

**Authors:** Wei Yu, Yukai Yang, Zhanhui Qi, Binbin Shan, Xiaolin Huang, Yan Liu, Heizhao Lin, Tao Li, Zhong Huang, Zhenhua Ma, Chuanpeng Zhou, Changping Yang

**Affiliations:** a Key Laboratory of South China Sea Fishery Resources Exploitation & Utilization, Ministry of Agriculture and Rural Affairs, Key laboratory of Fishery Ecology and Environment, Guangdong Province, South China Sea Fisheries Research Institute, Chinese Academy of Fishery Sciences, Guangzhou, People’s Republic of China; b Shenzhen Base of South China Sea Fisheries Research Institute, Chinese Academy of Fishery Sciences, Shenzhen, China; c School of Marine Sciences, Ningbo University, Ningbo, People’s Republic of China

**Keywords:** *Heniochus chrysostomus*, mitochondrial genome, phylogenetic analysis

## Abstract

We report the first mitochondrial genome sequences for the three band pennant fish, *Heniochus chrysostomus*. The whole mitogenome of *H. chrysostomus* was circular in shape and 16,650 bp in length. The mitogenome consists of 13 typical vertebrate protein-coding genes, 22 tRNA genes, 2 rRNA genes (12S rRNA and 16S rRNA), and 2 putative non-coding regions. Phylogenetic tree analysis revealed that *H. chrysostomus* was closely related to *Heniochus diphreutes*. This study will provide useful genetic information for future phylogenetic and taxonomic classification of Chaetodontidae.

*Heniochus chrysostomus* (Cuvier, 1831) belongs to the Family Chaetodontidae and the Order Perciformes, and it looks similar to the pennant coralfish but can be distinguished by the yellow tip to the snout and three distinctive diagonal black bands on the body (Parmentier et al. 2011). The species is distributed in Western India to Pitcairn Islands, north to southern Japan, south to Rowley Shoals, the south China sea, southern Queensland, and New Caledonia (Chen and Zhang 2015). In this study, complete mitochondrial genome of *H. chrysostomus* was characterized, which would provide genomic data for taxonomic resolution, population genetic structure and phylogenetic relationship.

Specimen was collected from Sanya, Hainan Province, Republic of China (N16°25'26″, E110°46'06″) in October 2019 and deposited in the Key laboratory of Fishery Ecology and Environment, Guangdong Province, South China Sea Fisheries Research Institute, Chinese Academy of Fishery Sciences, Guangzhou, China (voucher specimen number: HC-1). Total genomic DNA was extracted from the muscle tissue using the Aidlab Genomic DNA Extraction Kit following the manufacturer’s instructions (Aidlab Biotech, Beijing, China). The mitogenomes of *H. chrysostomus* was sequenced by next-generation sequencing (Illumina HisSeq 4000; Guangzhou JiRui Gene Technology Co. Ltd. China). Clean data without sequencing adapters were *de novo* assembled by the NOVOPlasty software (Dierckxsens et al. 2017). We compared the assembled genome with three confirmed sequences by PCR and Sanger sequencing methods to evaluate the single-base accuracy of the assembled mitochondrial genome. In this study, we obtained the complete mitochondrial genome of *H. chrysostomus*. Its mitochondrial genome is deposited in the GenBank under accession number MW136414. For a better understanding of genetic status and the evolutionary study, we focused on the genetic information contained in the complete mitochondrial genomes of the fish.

The complete mitogenome of *H. chrysostomus* was circular in shape and 16,650 bp in length with 16.24% G content, indicating an obvious anti-guanine bias commonly observed in other fishes (Cheng et al. 2012; Shan et al. 2016). The overall base composition was 28.64% of A, 25.47 of %T, 29.65% of C and 16.24% of G with a slight A + T bias (54.11%) like other vertebrate mitochondrial genomes. The mitogenome consists of 13 typical vertebrate protein-coding genes, 22 tRNA genes, 2 rRNA genes (12S rRNA and 16S rRNA), and 2 putative non-coding regions (control region and L-strand replication origin). All 13 protein-coding genes found in other vertebrates were also present in *H. chrysostomus* including three subunits of the cytochrome c oxidase (COI-III), seven subunits of the NADH ubiquinone oxidoreductase complex (ND1-6, ND4L), one subunit of the ubiquinol cytochrome oxidoreductase complex (Cyt b), and two subunit of ATP synthases (ATP6 and ATP8). The total length of those genes was 11,416 bp, accounting for 68.56%. Furthermore, as in other bony fishes, the mitogenome contained 22 tRNA genes interspersed between the rRNA and protein-coding genes. 14 tRNA genes were transcribed on the H-strand, whereas other 8 tRNA genes were oriented to the L-strand (Cheng et al. 2012; Li et al. 2013). Two of the 22 tRNA were determined for serine (UCN and AGY) and leucine (UUR and CUN), and one specific tRNA gene for the other amino acids. The tRNA genes varied in size from 65 bp to 75 bp.

To determine the taxonomic status of *H. chrysostomus*, we constructed the phylogenetic tree using the maximum likelihood method on the basis of the complete sequences of the mitochondrial genomes of 21 species (Yang et al. 2017), and we selected *Salvelinus malma* as the outgroup. The phylogenetic tree showed that the *H. chrysostomus* had the closer relationship with *Heniochus diphreutes* (Figure 1). The complete mitochondrial genome sequence of *H. chrysostomus* provided an important dataset for a better understanding of the mitogenomic diversities and evolution in fish as well as novel genetic markers for studying population genetics and species identification.

**Figure 1.**
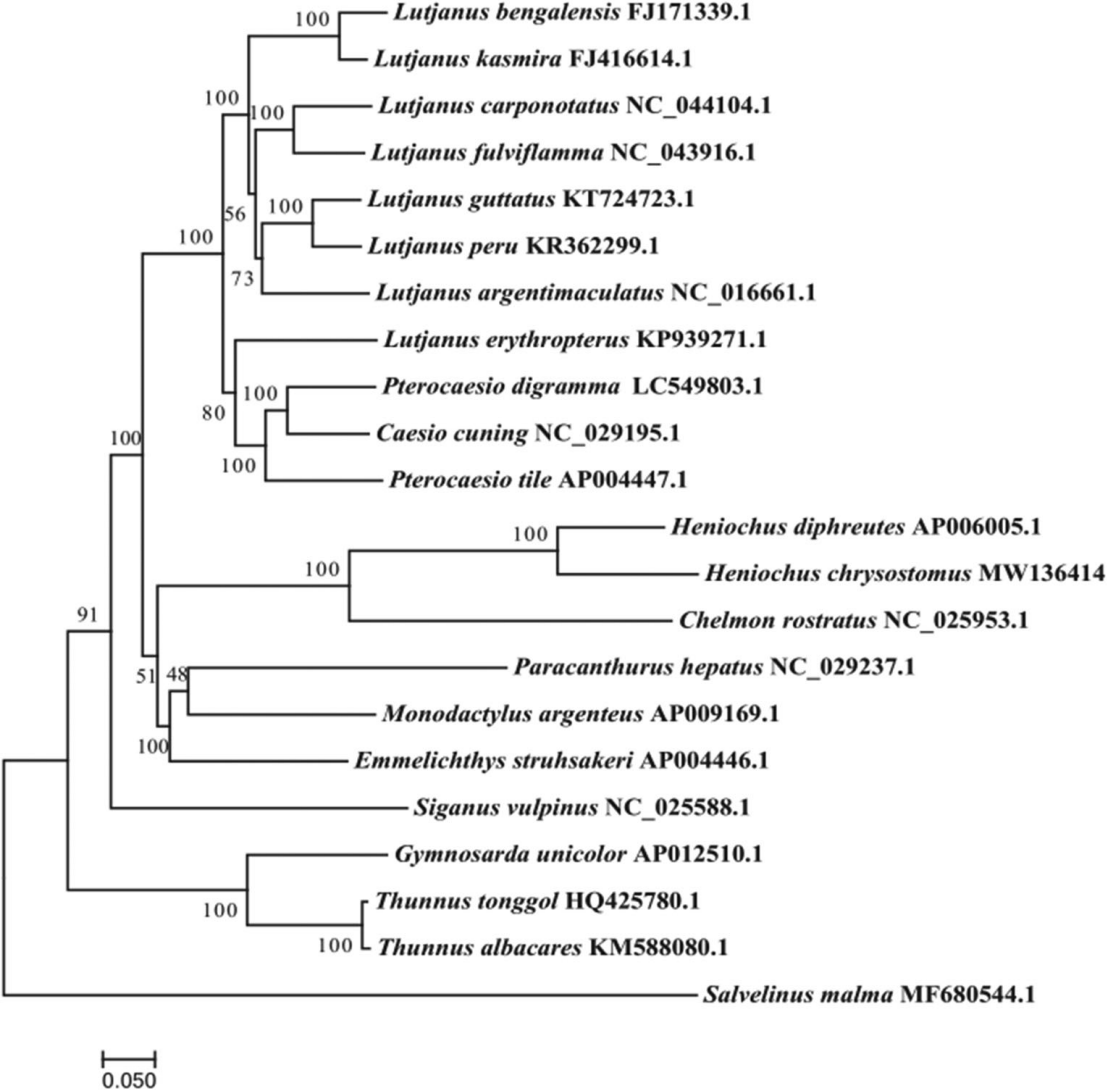
Phylogenetic tree of 22 complete mitogenomic sequences using the maximum likelihood method. Compared species: *Lutjanus bengalensis*, *Lutjanus kasmira*, *Lutjanus carponotatus*, *Lutjanus fulviflamma*, *Lutjanus guttatus*, *Lutjanus peru*, *Lutjanus argentimaculatus*, *Lutjanus erythropterus*, *Pterocaesio digramma*, *Caesio cuning*, *Pterocaesio tile*, *Heniochus diphreutes*, *Chelmon rostratus*, *Paracanthurus hepatus*, *Monodactylus argenteus*, *Emmelichthys struhsakeri*, *Siganus vulpinus*, *Gymnosarda unicolor*, *Thunnus tonggol*, *Thunnus albacares*.

## Data Availability

The data that support the findings of this study are openly available in [National Center for Biotechnology Information] at [https://www.ncbi.nlm.nih.gov/], reference number [MW136414].
